# Deep Species Distribution Modeling From Sentinel-2 Image Time-Series: A Global Scale Analysis on the Orchid Family

**DOI:** 10.3389/fpls.2022.839327

**Published:** 2022-04-22

**Authors:** Joaquim Estopinan, Maximilien Servajean, Pierre Bonnet, François Munoz, Alexis Joly

**Affiliations:** ^1^INRIA, Montpellier, France; ^2^LIRMM, Univ Montpellier, CNRS, Montpellier, France; ^3^AMIS, Université Paul Valéry Montpellier, Univ Montpellier, CNRS, Montpellier, France; ^4^AMAP, Univ Montpellier, CIRAD, CNRS, INRAE, IRD, Montpellier, France; ^5^CIRAD, UMR AMAP, Montpellier, France; ^6^LIPHY, Université Grenoble Alpes, Grenoble, France

**Keywords:** species distribution modeling, deep learning, image time-series, Sentinel-2, convolutional neural networks, remote sensing, macroecology, data science

## Abstract

Species distribution models (SDMs) are widely used numerical tools that rely on correlations between geolocated presences (and possibly absences) and environmental predictors to model the ecological preferences of species. Recently, SDMs exploiting deep learning and remote sensing images have emerged and have demonstrated high predictive performance. In particular, it has been shown that one of the key advantages of these models (called deep-SDMs) is their ability to capture the spatial structure of the landscape, unlike prior models. In this paper, we examine whether the temporal dimension of remote sensing images can also be exploited by deep-SDMs. Indeed, satellites such as Sentinel-2 are now providing data with a high temporal revisit, and it is likely that the resulting time-series of images contain relevant information about the seasonal variations of the environment and vegetation. To confirm this hypothesis, we built a substantial and original dataset (called *DeepOrchidSeries*) aimed at modeling the distribution of orchids on a global scale based on Sentinel-2 image time series. It includes around 1 million occurrences of orchids worldwide, each being paired with a 12-month-long time series of high-resolution images (640 x 640 m RGB+IR patches centered on the geolocated observations). This ambitious dataset enabled us to train several deep-SDMs based on convolutional neural networks (CNNs) whose input was extended to include the temporal dimension. To quantify the contribution of the temporal dimension, we designed a novel interpretability methodology based on temporal permutation tests, temporal sampling, and temporal averaging. We show that the predictive performance of the model is greatly increased by the seasonality information contained in the temporal series. In particular, occurrence-poor species and diversity-rich regions are the ones that benefit the most from this improvement, revealing the importance of habitat's temporal dynamics to characterize species distribution.

## 1. Introduction

### 1.1. Context

Understanding and mapping species distributions is a major topic in conservation biology (Pecl et al., 2017). Species distribution models (SDMs) have recently become a key instrument: over the last 20 years, 6,000 peer-reviewed studies were found with this keyword according to Araújo et al. (2019). These statistical algorithms learn the correlations between species presence records (and possibly species absence records) and some environmental predictors provided. Under certain modeling assumptions (Zurell et al., 2020), they can estimate species distribution by generalizing learned habitat preferences over time and space (Phillips and Dud́ık, 2008; Thuiller et al., 2009). A major issue for the use of SDMs concerns the ecological relevance of the predictive variables used (Fourcade et al., 2018). Furthermore, collecting appropriate data at a large scale is usually very challenging. Global bio-climatic variables do not systematically provide enough information to draw conclusions on a species, presence. Many other factors like species dispersal capacities (Monsimet et al., 2020) or shifts in land use actually come into play.

After having revolutionized computer vision, neural networks - and especially convolutional neural networks (CNNs) - are also increasingly recognized in ecology (Williams et al., 2009; Heikkinen et al., 2012; Botella et al., 2018; Brodrick et al., 2019). They allow identifying environmental patterns on images like tree crowns (Csillik et al., 2018) or forest type limitations (Wagner et al., 2019). Local environment spatial structure has already been proven to add relevant information to SDMs involving convolutional layers (Deneu et al., 2021b).

In addition, remotely sensed data can grasp key features of vegetation functioning and thus convey relevant insights on species habitats (Remm and Remm, 2009; Adhikari et al., 2012; He et al., 2015). Unmanned Aerial Vehicles (UAVs) allow finer and finer-scale coverage at local, regional, or even country scale (Kattenborn et al., 2020). Thanks to such imagery, the nature and spatial structure of ecosystems can be characterized and learned in SDM training. RGB and IR image patches around species occurrences (or digitized geolocated presence of species) are thus added to the environmental predictors, so as to include information on vegetation and land-use heterogeneity around the occurrences (Deneu et al., 2021a).

Satellite missions like Copernicus Sentinel-2 (S2) (Berger et al., 2012) now provide RGB and IR channels with fine spatial resolution and temporal revisit frequency worldwide (see Section 2.1.1), which can feed high-resolution, CNN-based SDM models. However, there is still much potential ahead for bringing together remote sensing and deep learning (Camps-Valls et al., 2021). Remote sensing datasets that are *(i)* readily available for deep learning applications and *(ii)* exploiting the spatial, spectral, and temporal dimensions of new satellite missions are still very few. For instance, among the twenty-three benchmark datasets implemented in *TorchGeo* (Stewart et al., 2021), only two encompass a temporal dimension. There is then an opportunity to build RGB+IR image time series around occurrences spread worldwide. By sampling S2 data for a whole year, prominence is given to the seasonal evolutions of the plants, habitats. These time series are capturing the signature of ecosystems phenology and productivity. Our hypothesis is that this information can significantly help SDM predictions.

### 1.2. Contributions

**Figure 1:**
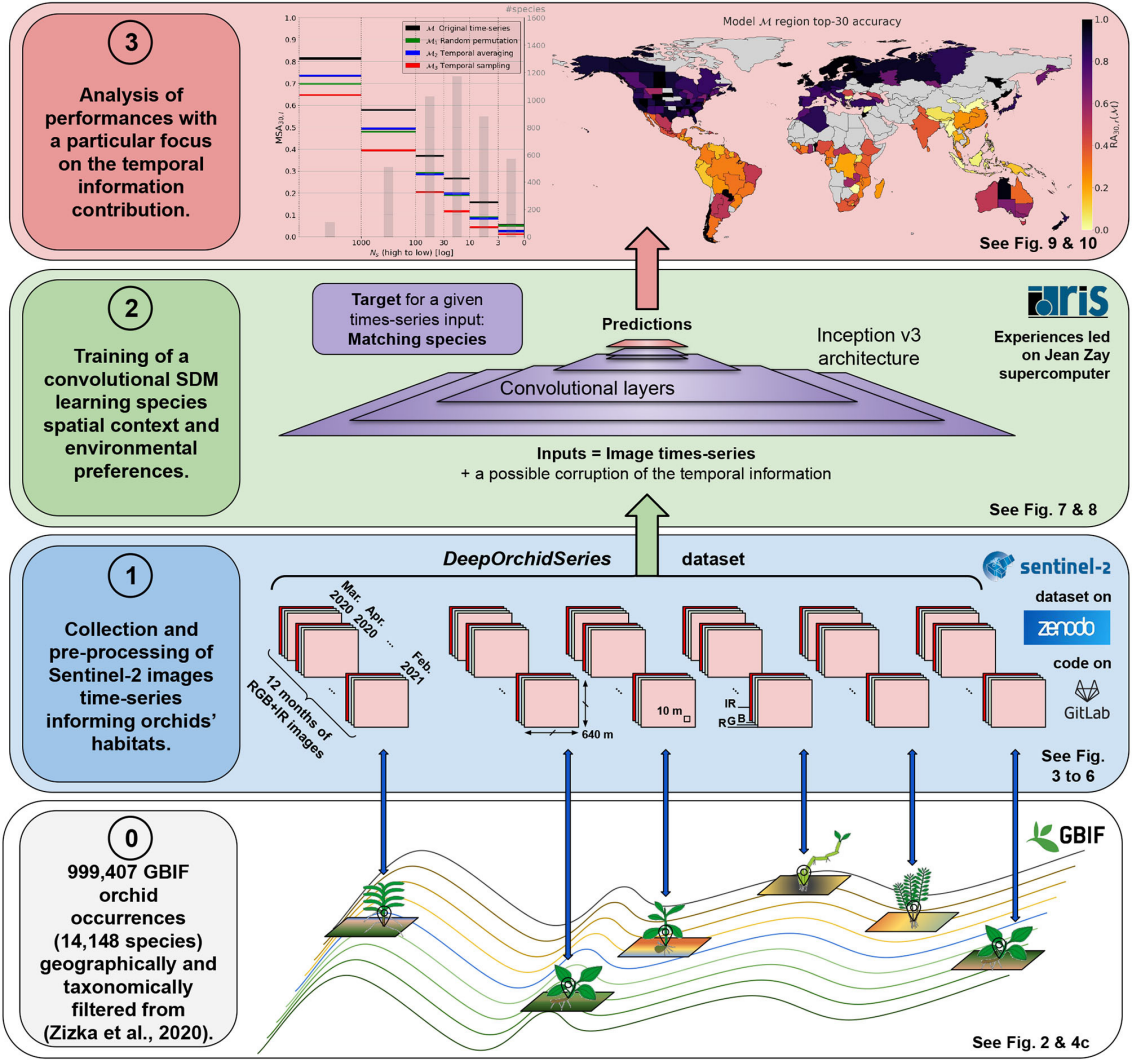
Visual abstract of the proposed method. Layer 0: The dataset introduced in this paper (*DeepOrchidSeries*) is based on a filtered set of GBIF occurrences (Global Biodiversity Information Facility) coming from the study of Zizka et al. (2021). Layer 1: Sentinel-2 image time series were collected around each occurrence geolocation, keeping least cloudy data tiles every month between March 2020 and February 2021. Images are made of 640 x 640 m RGB+IR channels with 10 m spatial resolution. The dataset is available on Zenodo and the method to create it on the Gitlab.inria platform. Layer 2: We then trained deep species distribution models (deep-SDMs) based on a convolutional neural network (CNN) (Inception v3) to capture the spatio-temporal context and environmental preferences of species. Next, we conducted experiments where the input temporal dimension was modified (randomized, averaged or sampled) so as to measure its contribution to model performance. Layer 3: the results are finally broken down into three main dimensions of analysis: species frequency in the dataset, bioregion, and species diversity in these bioregions. The analysis reveals that occurrence-poor species and diversity-rich regions are the ones that benefit the most from the improvement provided by the temporal information.

This paper contribution is 2-folds: First, we built a substantial and original dataset pairing nearly 1 million geolocated occurrences of the *Orchidaceae* family with satellite image time series. This dataset and the associated method scripts, released as open data and code, should be useful for conservation biologists and SDM users in general. To our knowledge, no similar ready-to-use dataset is already available. Second, we designed interpretability tests of the deep SDMs trained on this dataset in order to measure the importance of seasonal landscape variability in characterizing species habitat and niche. Figure 1 provides the visual abstract of our method.

## 2. Materials and Methods

### 2.1. *DeepOrchidSeries* Dataset

#### 2.1.1. Raw Input Data Description

##### Orchid Occurrences Dataset

The *Orchidaceae* family is of great interest because of its diversity (about 28,000 species estimated) and its aesthetic attractiveness (Chase et al., 2015). Orchids are of major concern for ecologists due to the numerous threats they are facing: habitat destruction, climate change, pollution, and illegal harvesting for horticulture and tourism industries (Wraith and Pickering, 2018). They are also considered as a relevant proxy of their ecosystem's health (Newman, 2009). Moreover, orchids are found on all continents in a wide range of habitats and they are blooming at very different altitudes. Such a range or environmental amplitude is difficult to achieve with other families, making the orchid family an excellent candidate for the purpose of our study (i.e., to measure the importance of seasonal variability in characterizing species habitat and niche).

Rather than collecting a new set of orchid occurrences to build our image time-series dataset, we decided instead to re-use the one introduced by Zizka et al. (2021). Their objective was different from ours (i.e., estimating the conservation status of orchids) but the set of occurrences they collected from GBIF meets two main criteria of interest for our study: (i) global scale and (ii) suitable data quality, thanks to several data filtering and cleaning processes (including the use of the R package *CoordinateCleaner* v. 2.0-9, Zizka et al., 2019). The complete process they use is summarized in the Supplementary Table 1 of their paper (Zizka et al., 2021). Another benefit of reusing (Zizka et al., 2021)'s occurrence data is to support the potential reuse of our deep-SDM for the automated assessment of the orchid's IUCN status. In the long term, this will improve the reproducibility and comparability of newly developed methods in this regard.

**Figure 2:**
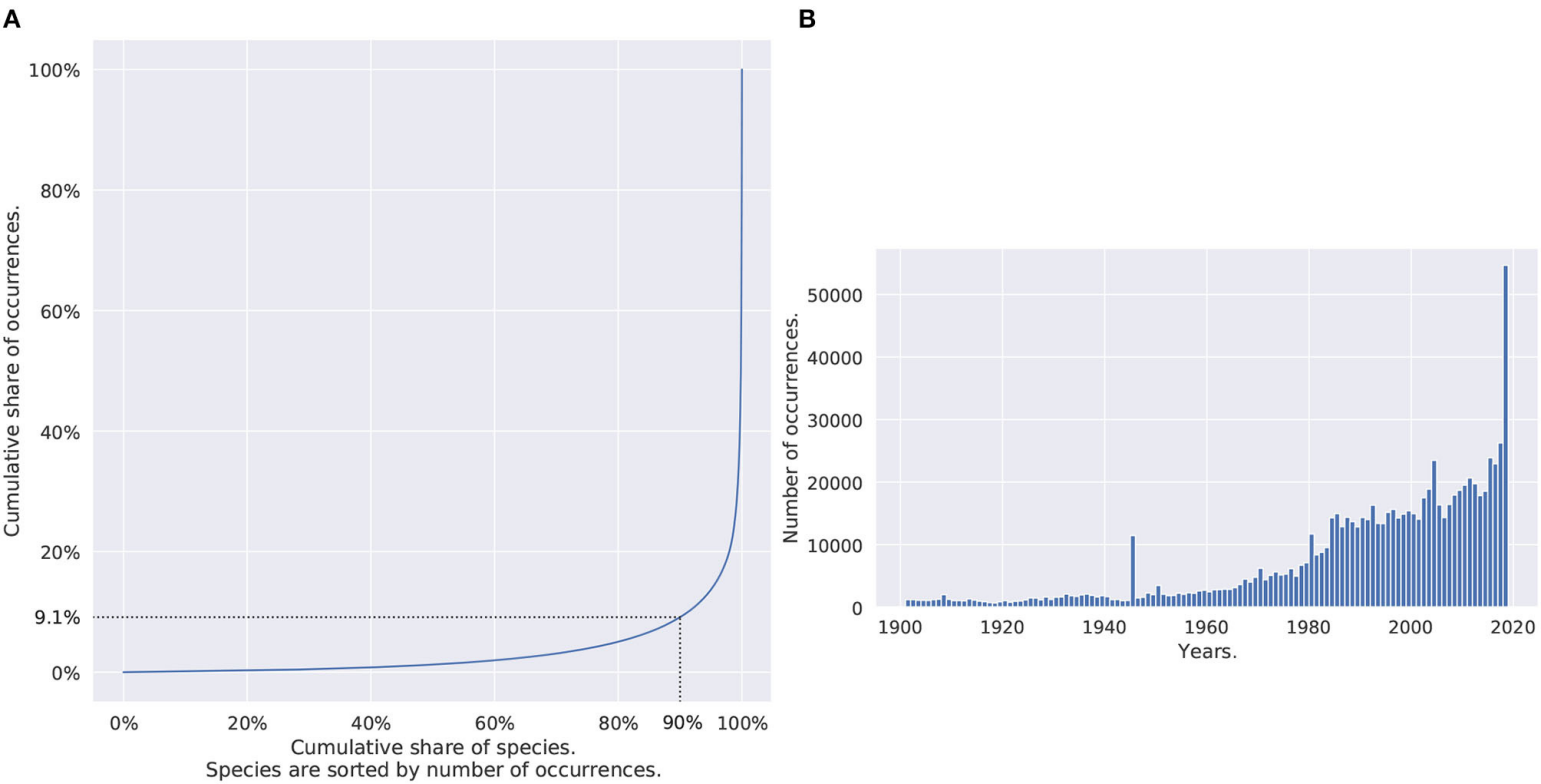
**(A)** Distribution of Occurrences of species. Species are ordered by frequency. The dotted lines are flagging that 90% of the species are only gathering 9.1% of the occurrences. **(B)** Temporal distribution of occurrences. The two graphs are based on all dataset occurrences.

In total, the dataset contains 999,407 occurrences of 14,148 species with 70 records per species on average, 4 in median, and 3,537 species (25%) with more than 13 observations. The (heavily-tailed) distribution of the number of occurrences per species is shown in Figure 2A (through a Lorenz curve). Figure 2B represents the temporal distribution of the occurrences in the dataset. Half of the observations dated from 1997, one quarter from 2010. A total of 14.6% of the set (145,641 occurrences) came with no timestamp at all. The oldest occurrence was from 1901 as a result of the filtering process that got rid of data records older than 1900. Only observations with a position uncertainty higher than 100 km were discarded. Perspectives and limits related to the use of such a large and imbalanced occurrence dataset will be discussed in the final Section 4.

##### Sentinel-2 Multispectral Images

Sentinel-2 multispectral data comes from two identical satellites in the same orbit but diametrically opposite to one another. Sentinel-2A was launched on 23 June 2015 and its counterpart Sentinel-2B on 7 March 2017. This satellite mission is part of the European Earth observation project Copernicus^1^, previously known as Global Monitoring for Environment and Security (GMES, Drusch et al., 2012). Thirteen channels from the visible to short-wave infrared are monitoring the planet, with 10, 20, or 60 m spatial resolution and a 5-day temporal revisit above any point on Earth. Additional satellites 2C and 2D are planned to ensure continuity in the coming years and the next generation of Sentinel-2 satellites are being prepared. We only kept four out of the thirteen channels, i.e., the three RGB channels and the Infrared (IR) channel (842 nm). These wavelengths are expected to convey the most relevant information about the environment (He et al., 2015) and are also the finer in terms of spatial resolution (10 m). The smallest geographic units downloadable *via* the *sentinelsat*^2^ API are 109.8 km × 109.8 km square data tiles in WGS84/UTM projection. They were defined following a military grid splitting Earth planisphere. The field square from a given satellite orbit at a given sensing time interval does not always cover a whole tile so that several products must be merged and cropped to get an image of the whole tile.

Data products are made available to the user at two distinct levels: Top-of-Atmosphere (TOA or 2C) and Bottom-of-Atmosphere (BOA or 2A). The important difference is the application of an atmospheric correction algorithm such as Sen2Cor (Louis et al., 2016; Ientilucci and Adler-Golden, 2019). Water vapor and other atmospheric components alter the satellite image caption with complex non-linear deformations. When and how atmospheric correction should be performed prior to exploiting remote sensing data depends on the desired information and thus the targeted application. About classification and change detection tasks, a recognized work from Song et al. (2001) advises performing simple corrections only when multi-temporal data is used. Otherwise, having both training and test sets from the same relative scale proved to be sufficient: no significant performance gain would result from the addition of an atmospheric correction step. A more recent article estimating the relation between sea surface salinity and Sentinel-2 Imagery with a neural network and 2,700 points obtained better results with TOA than BOA imagery (Medina-Lopez, 2020). On their specific application, they found that the atmospheric correction entailed information loss due to alteration of actual multispectral relationships. They also observed that the time and computational resources spared by using the level 2C products were an important element to consider. Using L1C products time-series, Rußwurm and Körner (2018) obtain state-of-the-art land cover classification performances. Level 2A products are not readily available at the global scale and, when needed, atmospheric corrections have in this case to be applied by users. Considering the conclusions of previous surveys and the large size of the targeted data, we decided to work with TOA products. Moreover, the atmosphere information could be valuable for our application and we suggest that deep-SDMs are capable of correctly learning without this additional filter.

#### 2.1.2. Dataset Construction

**Figure 3:**
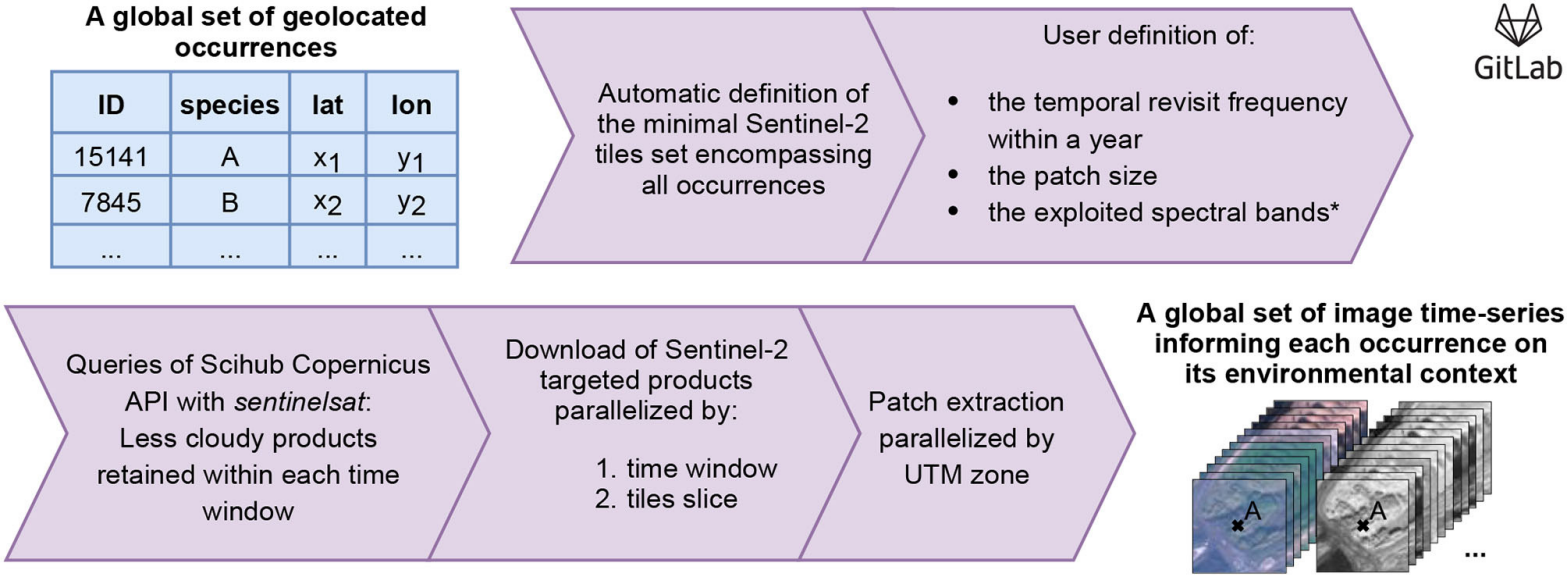
Creation workflow of *DeepOrchidSeries* dataset. Input is a set of geolocated occurrences, output gathers image time series informing on species habitat preferences. Code and details are available at https://gitlab.inria.fr/jestopin/sen2patch.

Figure 3 summarizes the workflow followed to obtain image time-series from a set of geolocated occurrences. The first step is to define the set of Sentinel-2 tiles containing all targeted occurrences, for which more details are provided in the *Global scale processing* paragraph. The second and third steps are used to define the *patch size* and the *time sampling strategy*, respectively. Our choices are presented in the two dedicated paragraphs hereafter. Finally, the last paragraph introduces our method to select the least cloudy S2 data.

We have furthermore considered only the four spectral bands available at 10 m resolution, but our workflow could be applied as well to bands at 20 m and 60 m resolution after a down-sampling step. Sentinel-2 queries and downloads were made with the Scihub Copernicus API^3^. We then extracted the patches by parallelizing the processing by UTM zone to gain speed. Code and details are available at https://gitlab.inria.fr/jestopin/sen2patch.

##### Global Scale Processing

**Figure 4:**
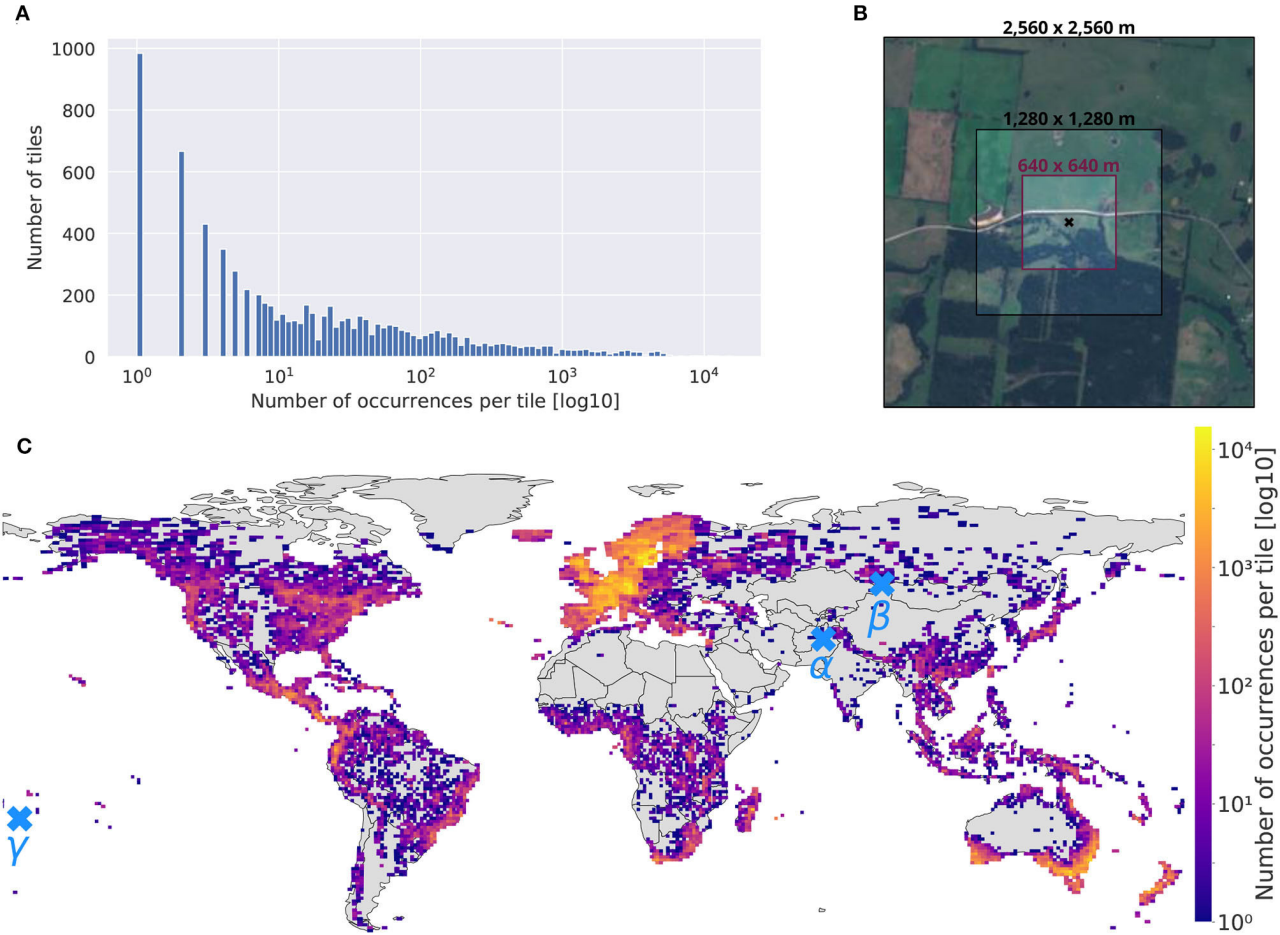
**(A)** Histogram of the number of occurrences per tile, **(B)** different patch sizes comparison around an occurrence located at (–39.883306, 144.050000), decimal degree system, **(C)** map of the selected tiles colored by the number of records contained (log10 scale). Three occurrences are located by α, β, and γ. **Figure 5** provides the three associated image time-series.

The first step consists then in defining the minimal set of Sentinel-2 tiles containing all our orchid observations. The *Sentinelsat* python API provides the option to query data by various geographical means, mainly, coordinates, polygons, tiles, or satellite orbits. However, querying the API on an occurrence-by-occurrence basis for a dataset containing nearly one million occurrences is counterproductive. It is much more efficient to first download the tiles containing occurrences and then extract them locally (as shown in Figure 4A for the histogram of the number of occurrences per tile). To do so, we implemented the following two steps:

- First, we created a dictionary linking each tile with its WGS84 geometry thanks to the *Sentinel-2 Level-1C tiling grid* provided by the ESA Sentinel-2 official portal^4^.
- Then, an iterative process on all occurrences was implemented, testing each time if the new observation is included in the union of the already retained tiles set. If not, a tile containing the occurrence location is downloaded and added to the set.

The final tiles set map is given in Figure 4C. It illustrates the full geographical scope of the dataset with 7,563 targeted tiles. A total of 50% of all land areas (Antarctica excluded) were included in the collected data. The color scale proportional to the number of observations per tile (with a log10-scale) further shows a geographic (or observation) bias in the occurrences set: Europe, south Australia, and New Zealand are gathering huge numbers of records.

##### Patch Size

The size of the patches associated with each occurrence is an important hyper-parameter to set. Patches should be large enough to contain the most relevant spatial information, but not too large to avoid introducing patterns that are too distant from the occurrence. They should also be large enough to compensate for the geographic imprecision of the occurrences (as shown in geolocation uncertainty distribution Supplementary Figure 1 and Wüest et al., 2020), but not too large to avoid computational issues. Considering all that constraints, our final choice was patches of size 640 m × 640 m (only powers of two were considered to optimize memory usage). Figure 4B illustrates three different patch sizes around an observation on an island of the South Australian coast. It shows that the 640 m × 640 m patch (40.96 ha) captures important landscape patterns around the record as well as potential threats due to surrounding land use.

##### Time Series Extent and Temporal Resolution

One of the main contributions of our study is to consider time series of satellite images rather than a single date image, with the objective of better characterizing the habitat of species. Two important parameters in this regard are the temporal extent of the series and its resolution. Here too, there is a compromise to be made. The extent and resolution must be high enough to capture important (spatio-)temporal patterns, but cannot be too high due to computational constraints. We finally chose a 1-year time series with a resolution of 1 month (i.e., twelve images, one per month).

**Figure 5:**
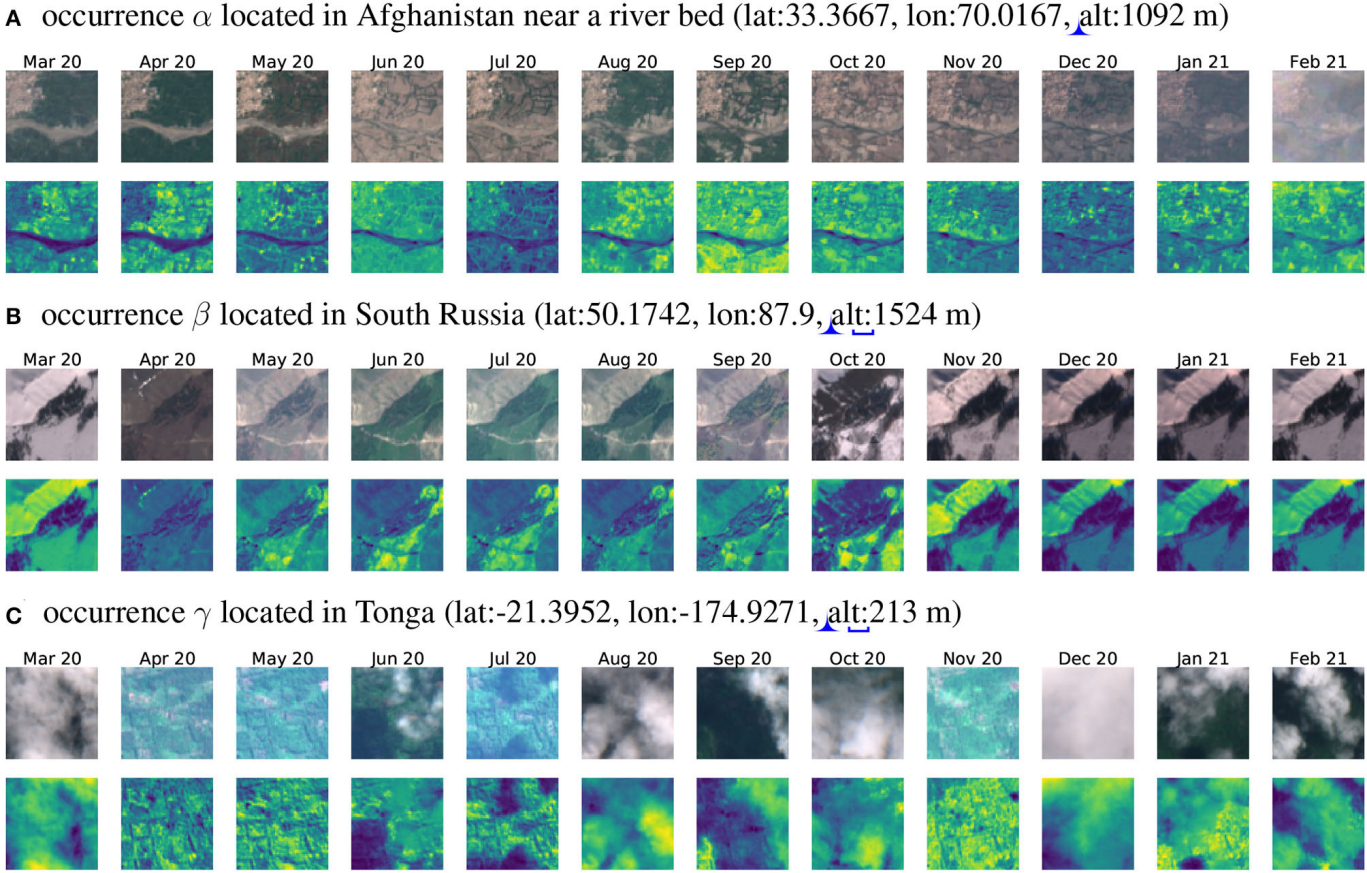
Image time series associated with the three occurrences located in Figure 4C map. RGB images are shown on the first line and IR patches on the second. **(A)** is almost cloud-free and globally normalized before visualization (i.e., all months are divided by TS maximum pixel), **(B)** is a cloudless time series with a strong environmental gradient because of snow presence and is normalized by frame (i.e., each month data is divided by month maximum pixel, only for visualization), **(C)** is an especially cloudy time series also normalized by frame.

Such 12-month time series allow grasping the main seasonal variations of the environmental and ecological context including vegetation phenology, yearly weather variations as well as landscape annual variations linked to human activity (e.g., agriculture). Noticeably, such seasonal variations are often neglected in SDMs devised at a global scale. Figures 5A,B show significant seasonal changes that can largely help models to differentiate species habitats. In Figure 5A, the tree cover greatly vary depending on the season and in Figure 5B snow covers the field half of the year. What if we only had 1 month of data? Environmental contexts would be characterized very partially and wrong inferences could be done on species ecological preferences (imagine having only one image covered by snow for Figure 5B). These examples illustrate the gain of ecologically relevant information when considering a 12-month image-series.

Another parameter to be set is the starting date of the time series. Ideally, it should be chosen so that the date of the occurrences is included in the 1-year period covered for the time series. There are various reasons in practice impeding a perfect match between the occurrences dates and the associated predictive data. To begin with, the Sentinel-2 satellite was launched only in 2015 so that older occurrences cannot be matched. Second, all occurrences do not come with a precise date, some having no date information at all. Third, some S2 tiles from the defined minimal set would have to be downloaded a huge number of times to inform all observations at different dates. Lastly, there is no simple and open access to data older than a rolling year on Copernicus Open Access Hub. Because of all that constraints, we finally chose a fixed period for all 12-month time series, with a starting date of 1 March 2020 and an ending date of 29 February 2021 (the choice of the recent period being linked to the temporal distribution of the number of occurrences, as shown in Figure 2B).

##### Data Selection Based on Cloud Cover

**Figure 6:**
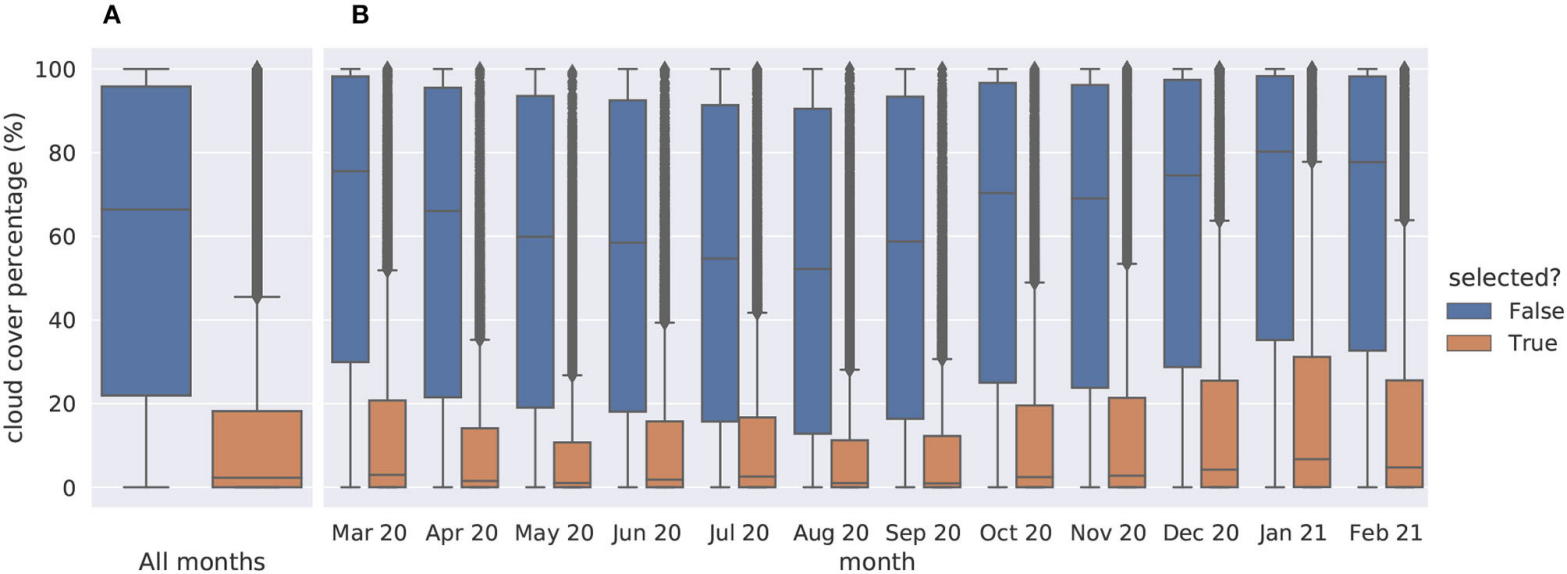
Cloud cover percentages of the 1,067,989 tested products, 180,747 (16.9%) selected against 887,242 (83.1%) dismissed. **(A)** all months taken together, **(B)** detailed by month.

Remote sensing data at RGB/IR channels are directly dependent on potential clouds covering the satellite's field of view. Fortunately, S2 products are including in their metadata the percentage of the scene view corrupted by cloud cover. Thereby when querying the *Sentinelsat* API over a given area and time window, one can ask to only keep the less cloudy products. The wider the chosen time window is, the more likely an almost cloud-free product will be available within. Based on this metadata, we selected the least cloudy S2 products within each month in the targeted time window. With this selection process, we expect the large majority of time series to be cloud-free like Figures 5A,B. Figure 6 provides an overview of the cloud coverage distribution in selected products compared to all available products in the queried time window. When, despite our efforts to select the least cloudy products, the obtained satellite data around an occurrence present many cloudy frames, it could nonetheless be interpreted as a piece of information contributing to the species, ecological niche. Furthermore, in this case, the environment structure can still be captured from clear scenes at other dates of the time series (see for instance April, May, and November 2020 on Figure 5C).

### 2.2. SDM Trained With Satellite Image Series

In this section, we describe the architecture and learning procedure of the deep-SDMs that we trained based on the *DeepOrchidSeries* dataset described above. Given an image time series as input, the model estimates orchids, *relative* probabilities of presence.

#### 2.2.1. Model Definition and Training Procedure

##### Model Architecture

The model used is an extended version of the Inception v3 (Szegedy et al., 2016) CNN. Inception networks are appreciated because of their capacity to grasp patterns -here environmental patterns- at multiple scales. It has been shown by Deneu et al. (2021b) that this architecture provides better species prediction performance than point neural networks, boosted trees, or random forests. We use this work to justify our choice of model. Nevertheless, testing other recent neural architectures specifically designed to deal with spatio-temporal data is an avenue to be exploited in the future, see the second perspective of the discussion. In particular, the performance gain was shown to be the most significant for rare species. In our context, the Inception v3 architecture was modified so as to accept not only RGB images but the full RGB+IR image time series. Our inputs are of size (*N*_*f*_, *N*_*x*_, *N*_*y*_) with *N*_*f*_ the number of features equal to 12 * 4 = 48 (12 months x 4 RGB+IR channels) and *N*_*x*_ = *N*_*y*_ = 64 (corresponding to 640 x 640 m quadrats at 10 m resolution). To speed up the training and regularize the model, batch normalization (Ioffe and Szegedy, 2015) was applied on the convolutional layer activations, just before the nonlinear ReLu function. Dropout (Srivastava et al., 2014) was finally used to prevent the network from overfitting (with a dropout probability of 0.5).

##### Model Loss

The models were trained using the LDAM loss (Label-Distribution-Aware Margin, Cao et al., 2019) designed for strong class-imbalance multi-class classification problems. In our context, it allows pushing upward rare species performance without deteriorating predictions on common species. The LDAM loss is a *label-distribution-aware* function that leads the model to an optimized trade-off between per-class margins. When considering two species only, say one rare and one common, the decision boundary drawn by this loss will be slightly shifted toward the common species in order to let the benefit of the doubt to the rare species (refer to Cao et al., 2019 Figure 1 for a meaningful scheme). The LDAM loss has been shown to perform very well in many deep learning benchmarks involving both a strong imbalance between classes and a high inter-class ambiguity.

##### Training Procedure

The models were fitted using stochastic gradient descent on multi-GPU nodes from *Jean Zay*, an IDRIS supercomputer^5^. They were trained during 70 epochs with a batch size equal to 64. The training process took around 100 h per model (with 8 gpus working in parallel).

Convolutional and linear layers weights were initialized from a truncated normal continuous random variable. The deferred re-weighting (DRW) training schedule associated with the LDAM loss was used. DRW is a vanilla empirical risk minimization (ERM) until a given epoch, here 65. Then, the training ends with a re-weighted loss and SGD steps with a re-normalized learning rate, both by batch species frequency. The learning rate was initialized to 0.1 and later decayed by a factor of ten at epochs 50 and 65. A trained model is approximately 600 MB.

#### 2.2.2. Performance Evaluation of the Model

##### Data Split

The *DeepOrchidSeries* dataset was split into three parts: (i) Training set (90%), (ii) Validation set (5%), and (iii), Test set (5%). Following the recommendations of Roberts et al. (2017), the split was done using a spatial blocking strategy that enables a more robust estimation of the performance of the model. The spatial blocks were defined in the spherical coordinate system according to a 0.025° grid, i.e., square blocks of 2.775 km at the equator. Splitting by block is important to impede the model from being validated or tested at locations very close to the training occurrences. In addition to the spatial blocking, we also used a stratified sampling strategy to ensure that any region of the world has a minimal number of blocks in the training set. We, therefore, used the WGSRPD level 2 regions (Brummitt et al., 2001). Within each region, we randomly sampled 90% of the blocks present and assign them to the training set. The remaining blocks were assigned to either the validation set or the test set (at random). Validation and test occurrences from species that were not in the training set were removed. Table 1 provides the number of occurrences and species in each set.

**Table 1:** Summary table of the number of occurrences and species in the training, validation, and test sets.

**Set**	**Training**	**Validation**	**Test**
#Occurrences	897,296	51,116	50,375
#Species	13,700	4,290	4,261

##### Evaluation Metrics

Our model being trained with a multi-class classification loss on presence-only data, its output is a categorical probability distribution of the form η_*s*_(*x*) = ℙ(*Y* = *s*|*X* = *x*) where *x* is the input tensor (i.e., an RGB+IR image time-series), *Y* the observed species and η_*s*_(*x*) is the estimated probability that the observed species is *s* conditionally to *x*. Because the output is a categorical probability distribution, we have that the sum of probabilities over all species is equal to one ($\overset{m}{\underset{s=1}{\sum }}\eta_{s}\left(x\right)=1$). To evaluate the model, we chose not to use pseudo-absences because of the bias induced by such methods (Phillips et al., 2009; Botella et al., 2020). Instead, we used a set-valued metric (Chzhen et al., 2021) to assess the quality of the species assemblage predicted by the model for a given input. Specifically, we chose the commonly used *top-k accuracy* as suggested in Botella et al. (2019). It measures the success rate of the model when it returns the top-k most probable species for any input *x*. More formally


(1) $$\begin{array}{r} \begin{array}{c} {\text{A}}_{k}=\frac{\sum_{i=0}^{n}{\text{A}}_{k}(i)}{n} \end{array} \end{array}$$

where *n* is the number of occurrences in the test set (or validation set) and


$$\begin{array}{l} {\text{A}}_{k}(i)=\left\{ \begin{array}{l} 1\text{ if }\eta_{y_{i}}(x_{i})\geq {\tilde{\eta }}_{k}(x_{i})\;\\ 0\text{ otherwise} \end{array}\right. \end{array}$$

with *y*_*i*_ the true species label of occurrence *x*_*i*_ and ${\tilde{\eta }}_{k}\left(x_{i}\right)$ the outputs of the model re-ordered in decreasing order of probabilities.

Because of the high-class imbalance of our dataset, a shortcoming of this metric applied on all test occurrences taken together (or *micro-average*, Sokolova and Lapalme, 2009) is that it gives far too much importance to the most frequent species over the less frequent ones. To compensate for this imbalance, it is preferable to use the *macro-average* version of this metric (Sokolova and Lapalme, 2009) consisting of first calculating the score of each species and then averaging the scores over all species. More formally, the macro-average top-k accuracy can be defined as


(2) $$\begin{array}{r} \begin{array}{c} {\text{MSA}}_{k}=\frac{\sum_{s=1}^{l}{\text{SA}}_{k,s}}{l} \end{array} \end{array}$$

where *l* is the number of species in the test and SA_*k,s*_ is the top-k accuracy for species *s* defined as


(3) $$\begin{array}{r} \begin{array}{c} {\text{SA}}_{k,s}=\frac{\sum_{yi\text{=}s}{\text{A}}_{k}(i)}{n_{s}} \end{array} \end{array}$$

with *n*_*s*_ the number of occurrences of species *s* in the test set. During the training phase of the model, the *macro-average top-k accuracy* (MSA_*k*_) is computed on the validation set every two epochs for *k* = 30. The model selected in the end is the one with the highest value.

To analyze the performance of the model according to the number of occurrences available in the training set, we also measured the macro-average accuracy on subsets of species categorized by a range of their number of occurrences. If we denote as *N*_*s*_ the number of occurrences of a species *s* in the training set, we can define as S_*I*_ = {*s*| *N*_*s*_ ∈ *I, n*_*s*_ > 0} the set of species in the test set having a number of training occurrences in a given interval *I*. The macro-average accuracy for a given interval *I* is then defined as


(4) $$\begin{array}{r} \begin{array}{c} {\text{MSA}}_{k,I}=\frac{\sum_{s\in {\text{S}}_{I}}{\text{SA}}_{k,s}}{\left|{\text{S}}_{I}\right|} \end{array} \end{array}$$

Another batch of experiences will focus on performances per geographic region. Spatial units are taken from the *World Geographical Scheme for Recording Plant Distributions* book (Brummitt et al., 2001). The level 3 division defines the *botanical countries* that we exploit. Performance per region *r* is denoted as RA_*k,r*_ and is defined as the micro-average top-k accuracy computed only on the occurrences encompassed in *r*:


(5) $$\begin{array}{r} \begin{array}{c} {\text{RA}}_{k,r}=\frac{\sum_{x_{i}\in r}{\text{A}}_{k}(i)}{n_{r}} \end{array} \end{array}$$

where *n*_*r*_ is the number of test occurrences in *r*. Regions with *n*_*r*_ fewer than 50 occurrences were excluded as statistically insignificant. Further, performance per region is compared with region's species diversity. Therefore, we computed the *diversity index* ${}_{}^{q}{\text{D}}_{r}$ of each region *r* according to the definition of Hill (1973) and Jost (2006). It is a quantitative measure of biodiversity combining, in a given region, species richness with species relative prevalence. The term prevalence is used instead of abundance to account for the observation bias in our data. Species richness corresponds to the number of distinct species observed (denoted L_*r*_). Species relative prevalence is the share of species occurrences compared to all region's observations: *p*_*s,r*_ equals $\frac{n_{s,r}}{n_{r}}$, with *n*_*s,r*_ the number of test occurrences from species *s* in *r*. The general expression of the region's diversity index is


(6) Dqr={(∑s=1Lrps,rq)11−q                   if q≠1       exp(−∑s=1Lrps,rln(ps,r))   if q=1

where *q* is a parameter weighting the trade-off between the importance granted to species richness (small value) vs. relative prevalence (big value). ${}_{}^{0}{\text{D}}_{r}$ results in regional species richness and ${}_{}^{1}{\text{D}}_{r}$ is the exponential of the Shannon entropy (Shannon, 1948). Performance per region is then averaged per category *I* on the diversity index and written as MRA_*k,I*_.

In the literature, the majority of studies involving species diversity use it as a *response* variable. They are focusing on its potential drivers like bio-climatic variables, topographic heterogeneity, or forest structure (Thuiller et al., 2006; Hakkenberg et al., 2016). Here, we exploit species diversity as an *explanatory* variable possibly explaining our model performances. In a similar manner, Emerson and Kolm (2005) defended that species diversity is a driver of speciation and (Dawud et al., 2016) examined its influence on soil carbon stocks among others.

#### 2.2.3. Interpretability Experiments: Quantifying the Contribution of Temporal Information

**Figure 7:**
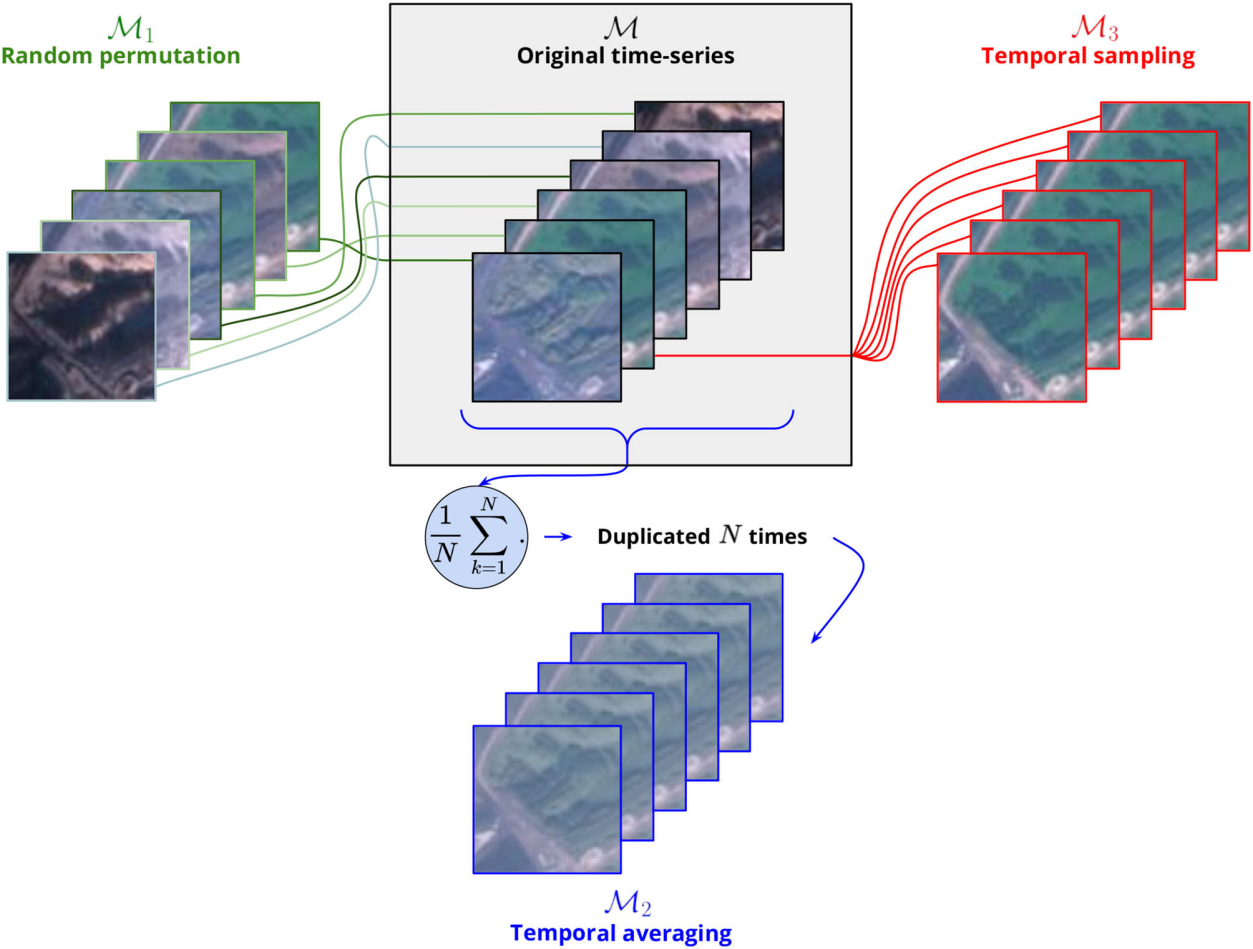
Scheme illustrating the three transformations applied to the input image time-series toward interpreting the contribution of the temporal information. Only 6 RGB images are depicted but these procedures are applied on the whole 12-month-long time series, IR channel included (here *N* = 6 but would normally equal 12). The image time-series *A* (black legend) corresponds to the original data, i.e., to the images stacked in chronological order. The image series *B*_1_ (green legend) is obtained by randomly permuting the original time series. The image series *B*_2_ (red legend) is made of 1 month picked at random and replicated *N* times. The image series *B*_3_ (blue legend) is constructed by averaging the 12 images of the original time series and replicating the resulting mean image *N* times. Please note that the same legend's colors will be used in the figures of the paper presenting the results of these experiments.

We designed several tests to analyze to what extent the trained model uses the temporal information contained in the image time series. The general principle is to transform the input data in order to suppress some information and to retrain a new model based on this transformed data. The comparison of the model deprived of information with the original model then allows quantifying the importance of the suppressed information. Figure 7 gives a comprehensive overview of the procedure detailed hereafter:

M **Original time-series**. This is the default original model where the input image time series are kept unchanged (stacked in chronological order). Here, the model can learn from the temporal dynamics present in the series. The filters learned by the Inception v3 model are themselves ordered feature maps time series of 12 months and are likely to capture spatio-temporal redundancies in the input data (e.g., seasonal variations of the environment or phenological patterns).
$M_{1}$ **Random permutation**. In this model, the 12 images of the original time series are randomly shuffled so that the model can no longer base its predictions on the actual temporal sequencing (Garnot et al., 2019). All input variance and spatial information remain nonetheless in the input. The filters learned by the Inception v3 model can neither be specialized by month nor can the model differentiate relations between months input. It actually learns from the block of 12 months considering them all equally. This procedure is comparable to the variable importance technique where a given input variable is randomized across samples to test how the model performs without its contribution. However, here, we do not randomize a given feature across samples, but features order independently for each sample.
$M_{2}$ **Temporal averaging**. In this model, the input image series are reduced to the mean over the 12 months replicated twelve times. Only the first moment of the distribution over the time dimension is kept and the model only "sees" a mean landscape averaged along the year. The objective here is to test to what extent a simple temporal averaging is sufficient to sum up most of the temporal variation. Each month contributes equally to the mean and the result is blurry. The variance between months has been totally removed. Ecological gradients of the different patch elements are reduced to their sum divided by twelve.
$M_{3}$ **Temporal sampling**. In this model, the input image series are reduced to only 1 month picked at random and replicated twelve times. The neural network is being provided with only a twelfth of the predictive data and is deprived of any temporal information.

Please notice that for each of the cases ($M_{1}$, $M_{2}$, and $M_{3}$), the data transformation is applied once on the whole dataset (including training, validation, and test set) before the model is trained and evaluated.

Model $M_{1}$ being deprived only of the months, order information, its comparison with Model M can be interpreted as a statistical test of the hypothesis that the composition of species depends on the existence of months specific features, in particular the ones resulting from yearly seasonality cycles. The comparison between M and $M_{3}$ can be interpreted as a test of the hypothesis that the species composition does or does not depend on any temporal variability. Model $M_{2}$ can be seen as an intermediate scheme where the temporal variability is summarized only by the mean of the distribution. Accordingly, the comparison between $M_{1}$ and $M_{2}$ allows assessing how useful statistical moments of a higher order than the mean are for characterizing the temporal variability.

To compare the performances of two different models, say M and $M_{i}$ with *i* ∈ {1, 2, 3}, for a given species *s* in the test set, we set a metric down called *relative performance change* of $M_{i}$ compared to M, defined as


(7) $$\begin{array}{r} \text{S}{\text{Δ}}_{k,s}\left(M,M_{i}\right)=\frac{{\text{SA}}_{k,s}\left(M\right)-{\text{SA}}_{k,s}\left(M_{i}\right)}{{\text{SA}}_{k,s}\left(M\right)} \end{array}$$

where SA_*k,s*_ is the top-*k* accuracy of species *s* (see Equation 3).

In the same manner that we defined the macro-average accuracy per category *I* on the species training set's number of occurrences, we can now consider the mean relative performance change per category between two models:


(8) $$\begin{array}{r} \text{MS}{\text{Δ}}_{k,I}(M,M_{i})=\frac{\sum_{s\in {\text{S}}_{I}}\text{S}{\text{Δ}}_{k,s}(M,M_{i})}{\left|{\text{S}}_{I}\right|} \end{array}$$

Relative region performance change $\text{R}{\text{Δ}}_{k,r}\left(M,M_{i}\right)$ is also calculated as $\frac{{\text{RA}}_{k,r}\left(M\right)-{\text{RA}}_{k,r}\left(M_{i}\right)}{{\text{RA}}_{k,r}\left(M\right)}$. This measure is averaged per category *I* on the diversity index as well and is represented by $\text{MR}{\text{Δ}}_{k,I}\left(M,M_{i}\right)$.

When computing $\text{S}{\text{Δ}}_{k,s}\left(M,M_{i}\right)$ (resp. $\text{R}{\text{Δ}}_{k,r}\left(M,M_{i}\right)$) between M and $M_{i}$ models for a given species *s* (resp. a given region *r*), it is beforehand necessary to make sure that the denominator, ${\text{SA}}_{k,s}\left(M\right)$ (resp. ${\text{RA}}_{k,r}\left(M\right)$), is not null. It can sometimes be when the model M fails to predict the correct label for all *s* occurrences (resp. all occurrences in *r*). In this case, no performance change can be calculated since it is already null. Species *s* (resp. region *r*) is then removed from the calculation of the mean performance change by categories on species training set number of occurrences (resp. on regions diversity index). This is why there is a drop of support between **Figure 9** (resp. **Figure 10**) left and right graphs, i.e., there are fewer species (resp. regions) encompassed in the categories, as indicated on the horizontal axis. This effect is a lot more important on the support of the species mean performance change than on the region's one. To sum up, relative performance change cannot be calculated for species or regions having already the lowest possible score with the whole temporal information. They are in that case discarded from the mean performance change calculation.

## 3. Results

### 3.1. Model Validation and Performance

**Figure 8:**
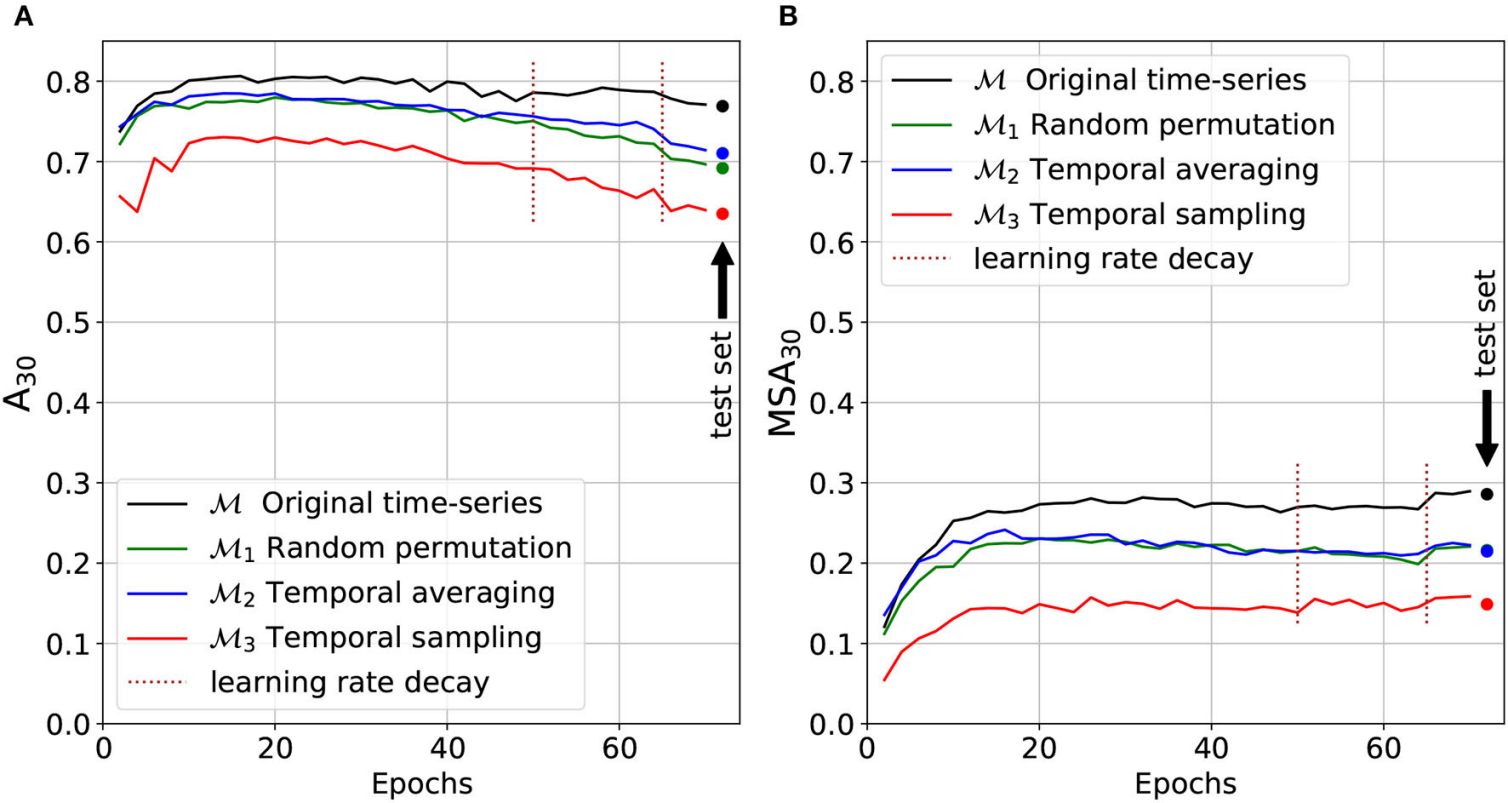
Micro **(A)** and macro **(B)** average top-30 accuracy on models validation and test sets. Micro-average results tend to represent common species whereas macro-average performances are more representative of rare species.

The top-30 and macro-average top-30 accuracy of the four models (M, $M_{1}$, $M_{2}$, and $M_{3}$) are presented on Figure 8 (at each epoch of the training phase for the validation set and on the test set for the final selected model). Due to the long-tail distribution of species occurrences (Figure 2A), the top-30 accuracy A_30_ is representative of the performance on the most common species whereas the macro-averaged top-30 accuracy MSA_30_ is more representative of the performance of the rare species. The final increase in the MSA_30_ score at epoch 65 is due to the DRW optimizer previously described: re-weighting the loss toward training's end enables a boost on rare species performances (Cao et al., 2019). The top-30 accuracy A_30_ tends to slightly decrease after the first quarter of the training phase. Our hypothesis is that this is mainly due to the use of the LDAM loss: as the training goes by, the models are reaching a better estimation of rare species ecological niche and tend to predict them more often to the detriment of common species that were chosen by default.

The model M trained and tested with the original time series provides better results than the three other models deprived of temporal information. M is the only one where the temporal dynamics are undamaged and hence fully exploitable to statistically draw predictions. The macro-average top-30 accuracy is 0.286 for the unaltered model M, against 0.216 for $M_{1}$ trained on shuffled data, 0.215 for $M_{2}$ trained on the yearly mean, and 0.149 for $M_{3}$ trained on a single random month.

The following analyses can be made of these results:

1. The strong performance decrease between M and $M_{3}$ shows that the temporal information contained in the time series is a key factor of the predictive performance. For most species, it appears to be as important as the spatial information alone (cf. macro-average accuracy plot MSA_30_).
2. The comparison between M and $M_{1}$ shows that the decisive temporal information is largely related to the order of the images in the time series, i.e., to the months, specific features captured by the model (such as the ones resulting from yearly seasonality cycles).
3. The comparison between models $M_{1}$ and $M_{2}$ shows that their performances is almost identical (cf. MSA_30_ plot). This means that the decisive information related to the unordered temporal variability can be synthesized efficiently by the mean of the time series. In other words, higher order statistical moments of the temporal dynamic independent from the time of the year are likely to be useless for predicting species composition (e.g., the standard deviation of acquisition noise).
4. The comparison between models $M_{1}$ and $M_{3}$ shows that the decisive temporal information is also largely explained by the unordered temporal variability of the images (typically due to some stochastic processes independent from the time of year).

### 3.2. Results by Number of Species Occurrences

**Figure 9:**
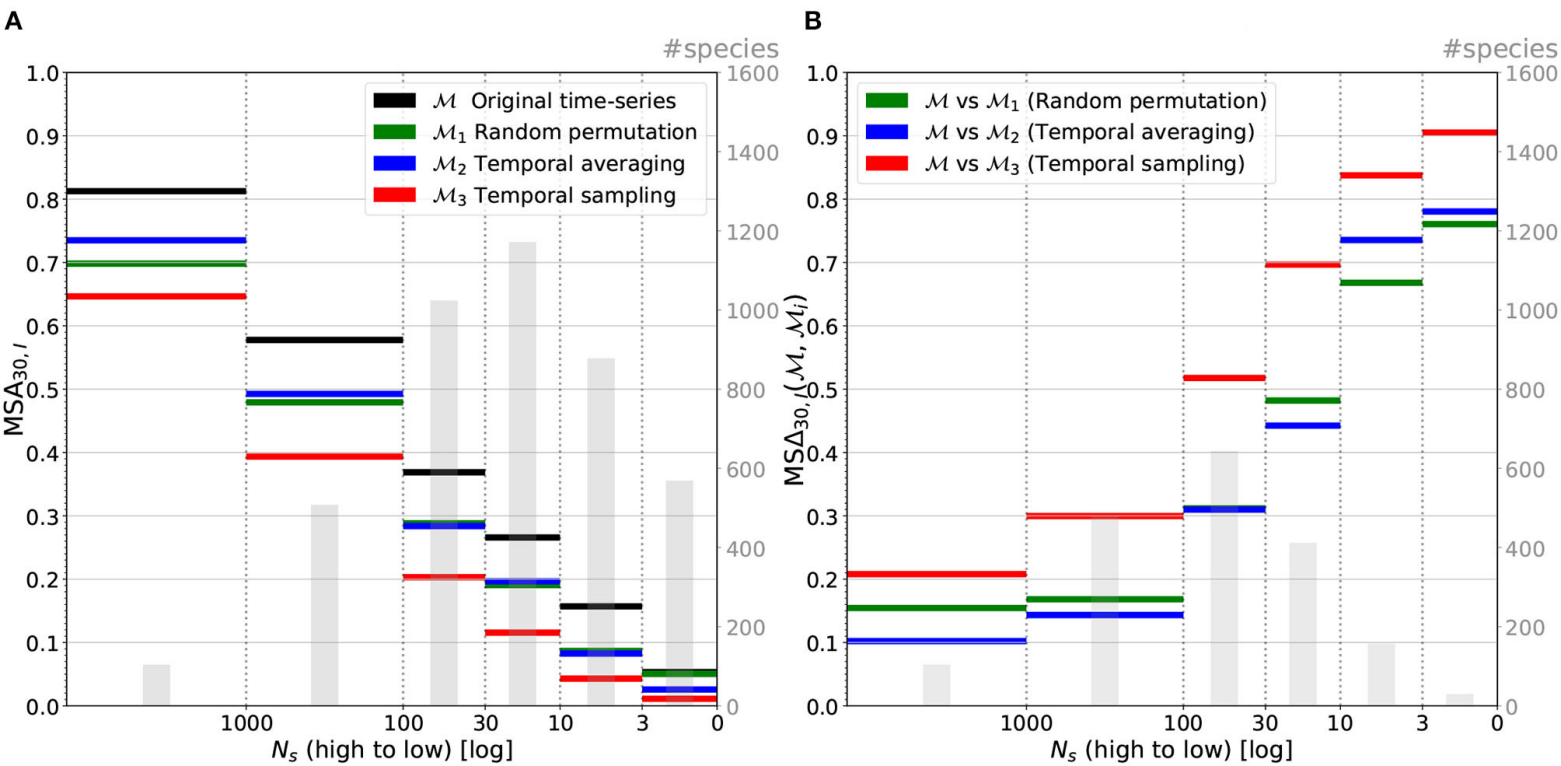
Macro-average top-30 accuracy **(A)** and relative top-30 accuracy change **(B)** averaged per category of the number of species occurrences in the training set. All models, performances are following the drop of *N*_*s*_ when relative performance changes are inversely proportional to it.

Figure 9A displays the performance of the four models as a function of the number *N*_*s*_ of species occurrences in the training set (cf. equation 4). Not surprisingly, we can observe that the accuracy of the model is positively correlated with the number of occurrences. The more the occurrences in the training set and the better the top-30 accuracy. It should be noted, however, that the performance on the rarest species remains much better than that of a random predictor. Species having between 3 and 10 occurrences, for instance, are predicted in the set of the top-30 most probable species in 17% of the cases. A random predictor over the 13,700 species of the training set would have a top-30 accuracy below 0.22%.

Figure 9B displays the mean relative performance change between the unaltered model M and the three models $M_{i}$ (*i* ∈ {1, 2, 3}) as a function of the number of species occurrences (as shown in Equation 8). It shows that the relative performance drop is inversely correlated with the species, number of occurrences. In other words, the rarer the species (in the data), the higher the performance gain obtained thanks to the temporal information. This can be explained by the fact that this is precisely on rare species predictions that the room for improvement is the bigger, as depicted on graph Figure 9A. The use of time series thus makes it possible to compensate for the lack of occurrence data by increased knowledge of the temporal dynamics of the environment.

### 3.3. Results by Region and Regional Diversity Index

**Figure 10:**
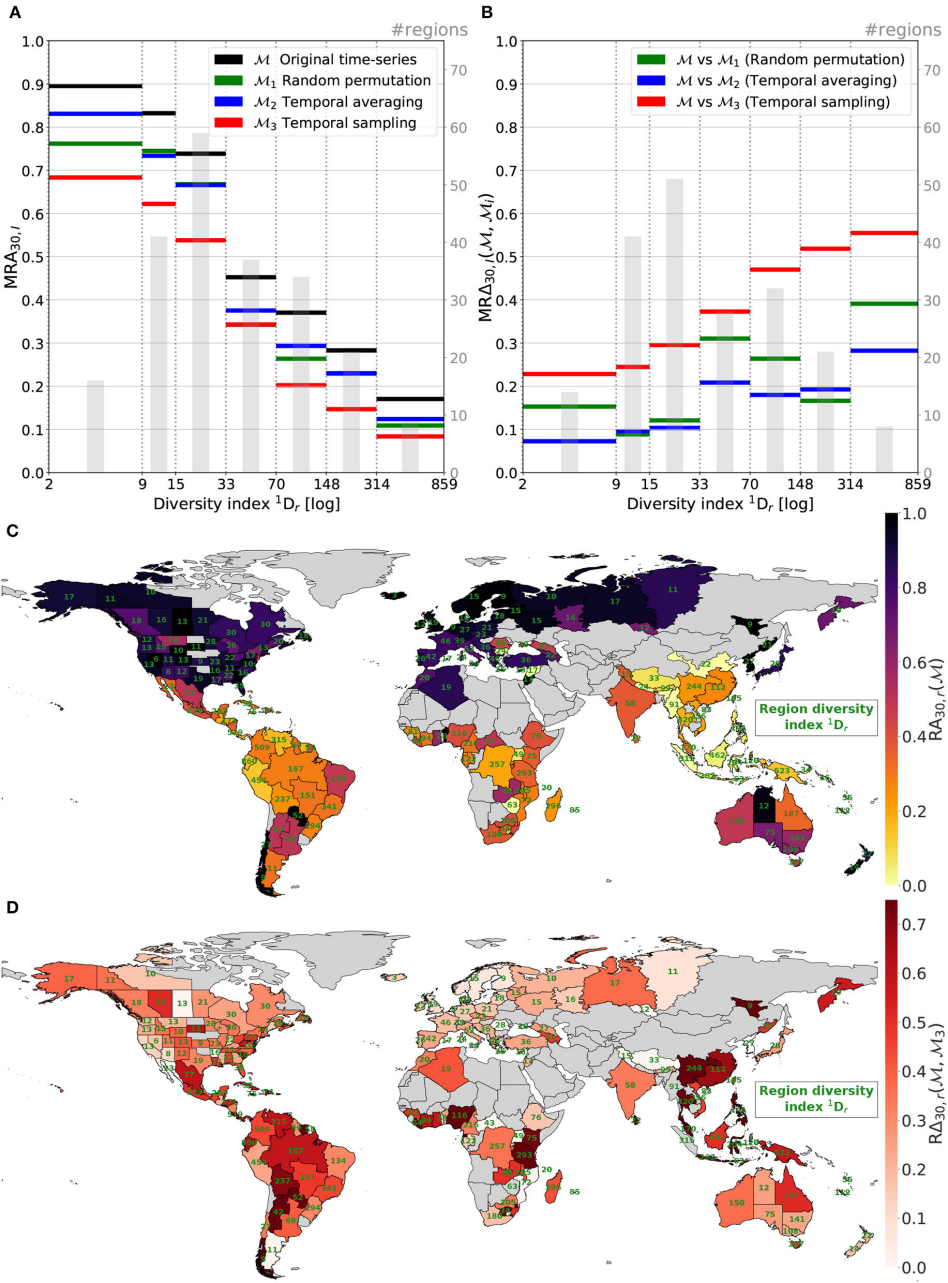
Region top-30 accuracy **(A)** and relative top-30 accuracy change **(B)** averaged per cat. of ${}_{}^{1}{\text{D}}_{r}$. Map **(C)** presents region top-30 accuracy with ${}_{}^{1}{\text{D}}_{r}$ indicated in green. Map **(D)** illustrates spatial decreases in performances when comparing $M_{3}$ to M, i.e., without/with the temporal information.

Figure 10 displays all results related to the regional analysis of our models.

The first sub-graph Figure 10A shows that the predictive performance of the four models is negatively correlated with the regional diversity index. Regions with small diversity indexes ${}_{}^{1}{\text{D}}_{r}$ are the ones where the model predictions are the better. On the contrary, regions with high diversities show the models achieve poor performance. With *q* = 1, the diversity index equals the Shannon entropy exponential. This measure strongly depends on species richness. Hence, areas with high diversities are where there is a lot of possible different orchids. This means many possible classes for the models and a high risk of confusion between species with similar environmental preferences. Moreover, these areas are often including a lot of rare species and/or are still poorly observed. Regions with low ${}_{}^{1}{\text{D}}_{r}$ values are regions with relatively low species richness and tend to encompass common species that the models are predicting well (as shown in Figure 9A).

The second sub-graph (Figure 10B) displays the relative performance change when comparing the model M to $M_{i}$ models, as a function of the regional diversity index. The most obvious trend is the red curve: when totally deprived of the habitat temporal dynamics, predictions on most diverse regions are proportionally more impacted than on low diversity regions. The tendency is more irregular for $M_{1}$ and $M_{2}$ but is globally valid too. It implies that, similar to rare species in Figure 9B, the temporal information especially benefits highly diverse areas. The enlightenment of this tendency also is that this is where the room for improvement is the largest. Models especially take advantage of further temporal information to progress on hard tasks. Supplementary Figure 2 presents the results of the same experience but with categories formed on regions' the number of occurrences in the training set *N*_*r*_, the total number of occurrences entailed in region *r* during training. Unlike Figures 10A,B, no tendency can be drawn. It reaffirms our idea that it is region's diversity that is driving results spatially and not only the observation bias.

The map displayed in Figure 10C depicts the top-30 accuracy per region achieved by the model M (i.e., the unaltered model with original time-series). A clear difference in performances can be observed between the southern and northern hemispheres. Looking at regions' diversity index ${}_{}^{1}{\text{D}}_{r}$, written in green on the map, allows a better understanding of this gap. Northern regions (especially northern Europe) are presenting fewer species and are well sampled whereas regions around and below the equator (Australia excepted) are a lot more diverse and still insufficiently observed. Models, average performances are actually quite consistent on the Earth parallels. This map is the direct illustration of the Figure 10A black curve.

Finally, map Figure 10D shows that where the loss of the temporal information impacts the more the performances. It corresponds to red curve of Figure 10B when the model trained with only one randomly picked and duplicated data month is compared to the reference model trained with full time series. Relative performance decreases in very diverse regions like southern China or Bolivia are really pronounced. On the contrary, performances in countries with low orchid diversities and well-observed like Norway of Finland are relatively spared by the input reduction.

### 3.4. Statistical Tests

A t-test between M and $M_{1}$ species micro-average accuracies ${\text{SA}}_{30,s}\left(M\right)$ and ${\text{SA}}_{30,s}\left(M_{1}\right)$ does confirm that results are notably different (p-value of 5e−42). The same conclusion arises from the comparison of the average top-30 accuracy per region: ${\text{MRA}}_{30}\left(M\right)=0.591$ with ordered data against ${\text{MRA}}_{30}\left(M_{1}\right)=0.509$ without, a *p*-value of 3.5e−9. This confirms that the order of the images in the time series does matter and that providing the data stacked in chronological order leads to significantly better performances than when providing data in random order.

### 3.5. Model Evaluation Regarding Time and Spatial Data Mismatches

**Figure 11:**
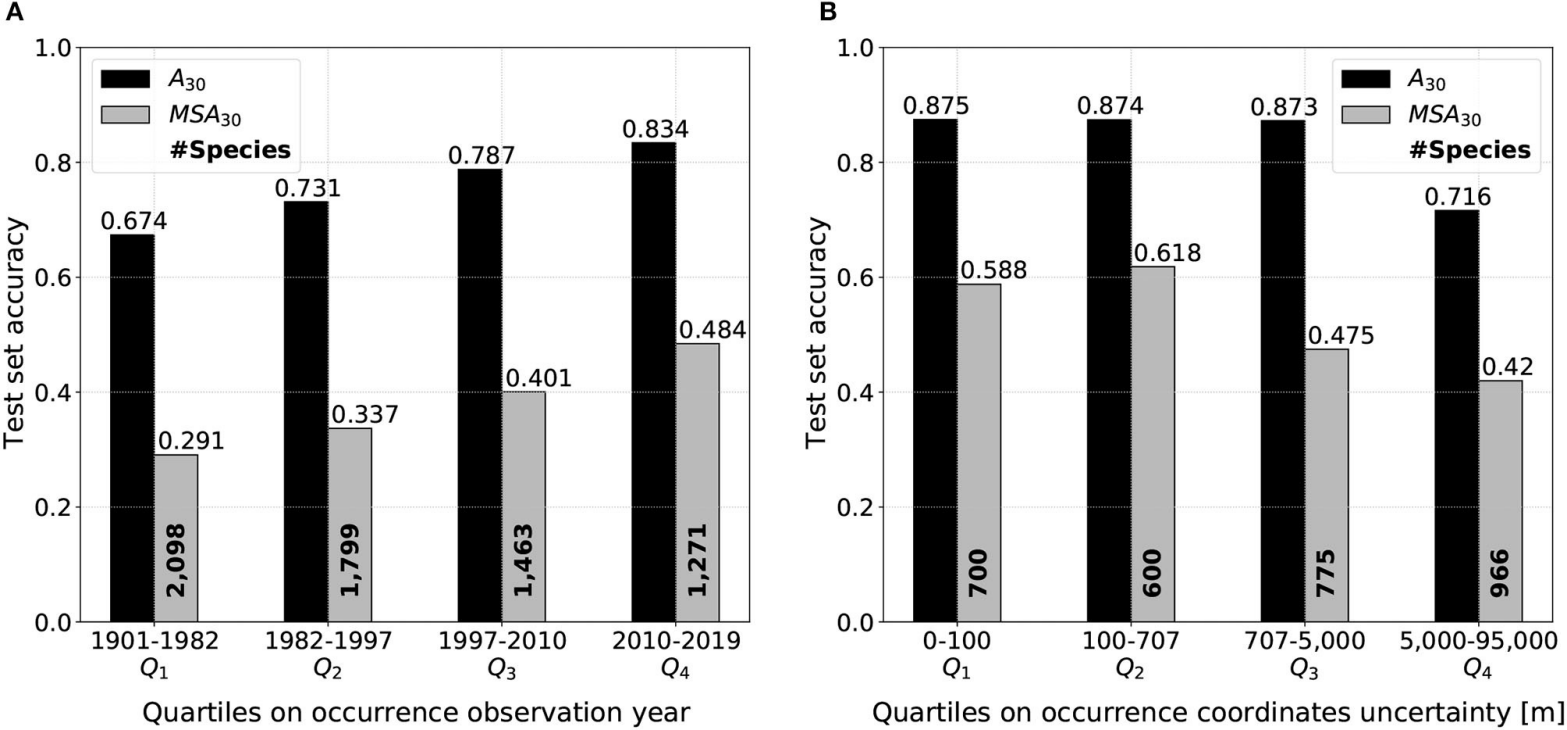
Model performances on the test set divided by quartiles *Q*_*i*_ on **(A)** occurrence observation year and **(B)** occurrence coordinates uncertainty. The test accuracy is higher on more recent observations and on observations with reasonably low coordinates uncertainty.

Figure 11A reveals a marked gradient of performance depending on test occurrence observation year. This analysis discarded 15% of the 50,375 test occurrences presenting no observation date information. Each quartile includes approximately 11,000 points. Both micro and macro top-30 accuracy seem to be linearly correlated to the occurrence observation year quartile. The linear behavior is confirmed when choosing a division with a thinner percentile. Top-30 performances on the last quartile 2010-2019 are impressive: 0.834/0.484 of micro/macro average accuracy. When cutting the test set data at the median 1997, i.e., considering separately the oldest and the most recent half of test observations, performances are of 0.703/0.281 (oldest half) and 0.811/0.409 (most recent half). Moreover, it should be noted that all macro-average performances calculated on the test set's subsets are comparatively higher than overall performances because less distinct species are considered (as shown in Figure 11 species number in bold, against 4, 261 in the entire test set).

Figure 11B focuses on the influence of test occurrence coordinates uncertainty on model performance. Test set is divided by quartiles on the studied variable, likewise Figure 11A. In total, 31% of test observations do not include any information on coordinates uncertainty and are consequently put aside. Each quartile contains approximately 9,000 observations. Micro-average top-30 accuracy is identical on the first three quartiles and only drops when uncertainty is higher or equal than 5,000 m. Macro-average top-30 accuracy is similar when uncertainty is kept under 707 m, i.e., for the first two quartiles only (it is even slightly higher for the second one). Then, the macro-average performance goes a step down starting from the median of 707 m. Both micro and macro average performance are severely diminished when coordinates uncertainty is superior or equal to 5 km.

## 4. Discussion

### 4.1. SDMs and Satellite Data

Remote sensing is an invaluable source of predictive features for SDMs and more widely for deep learning based earth observation applications (He et al., 2015; Zhu et al., 2017; Borowiec et al., 2021). Combined together, they offer a key opportunity in monitoring biodiversity facing climate change (Randin et al., 2020).

Species distribution models coupled with remote sensing data are often exploiting the widespread vegetation indexes Enhanced/Normalized Difference Vegetation Index (EVI or NDVI, Bannari et al., 1995). These indices are computed from satellite channels and are intended to reflect vegetation properties. The NDVI is said to assess photosynthetic activity and productivity (Pettorelli et al., 2011). Texture measures derived from satellite EVI were proven adapted to map habitat heterogeneity and bird species richness patterns (Farwell et al., 2020).

The WorldClim variables (weather station data interpolated with satellite-derived covariates, Hijmans et al., 2005; Fick and Hijmans, 2017) certainly are the most widely used global SDM predictors (Nogués-Bravo, 2009; Svenning et al., 2011). This bio-climatic data approaches habitats, annual trends (e.g., annual precipitation) and seasonalities (e.g., temperature annual range and standard deviation). Contrary to our 1-year *DeepOrchidSeries* dataset, here, the variables are averaged across several decades. Comparing the predictive power of these classic predictors (possibly completed with a land-cover raster) to our Sentinel-2 data will be the focus of future work.

Species distribution models and remote sensing data can also help rare species detection by capturing the biophysical conditions driving their distributions (Cerrejón et al., 2021). Recent studies have successfully leveraged the spatial structure of satellite images as input to CNN-based SDMs (Deneu et al., 2021b). Trained on fine-scale tensors, these models were proven able to learn and cluster species ecological preferences like annual mean temperature (Deneu et al., 2021a).

Regarding the use of the temporal dimension of satellite data in SDMs, few studies actually take advantage of it as underlined in Randin et al. (2020). In this regard, we can cite (Cord and Rödder, 2011) who tried in 2011 to include EVI seasonality information in their SDMs inputs. Their study was however on a totally different range than us since they focused on eight Mexican anurans and used one-dimensional predictors.

### 4.2. Benefits of Deep-SDMs Trained on Remote Sensing Image Time-Series

The main outcome of our study is that using time-series of satellite images significantly improve Deep-SDM performance, in particular for rare species and in most diverse regions, supporting the interest of the approach for conservation science. Rare species are almost always threatened due to few occurrences means, without conservation measures, and greater extinction risk. Moreover, the world's most diverse regions include nearly all undiscovered species (Joppa et al., 2011). Better knowledge of the ecological niche of rare or little-prospected species should foster more appropriate and effective conservation measures to ensure their survival.

We collected time series of remote-sensing images to grasp the temporal variation in habitat properties. Our results confirm that this information is of high value to capture species, ecological niches and potential distributions. Our time series are also providing SDMs with the spatial structure of species habitats, a key information to enhance predictive performances (Deneu et al., 2021b).

Recent satellite missions offer both high temporal revisit frequency and high spatial resolution at the global scale, supporting the use of such data for niche modeling. The use of even more intensive remote sensing data, e.g., all products without any selection by month or on a wider time window, would probably allow even better estimation of ecological niche. That said, the Sentinel-2 data curation we devised here represents a good trade-off to acknowledge the phenology of orchid habitats at a broad spatial scale. Trying to avoid as much as possible clouds on selected images was also a sensitive point in our dataset creation workflow. A thinner temporal resolution would have resulted in richer time-series, but also a higher number of cloud frames. The question of whether the presence of clouds is in itself a piece of relevant information for characterizing the environment was not addressed in our study and remains nonetheless an open question.

### 4.3. Comparison With Other Open Remote Sensing Datasets for Deep Learning

Remote sensing datasets for deep learning applications are currently gaining much interest and are more and more accessible. The very recent launch of *TorchGeo* (Stewart et al., 2021), a Python library to easily handle geospatial datasets in the PyTorch environment, illustrates the recent and still ongoing progress. However, the available datasets remain currently few and the temporal information provided by satellite revisits is almost never used (Sumbul et al., 2019). The available datasets are mostly used for land-cover classification (Helber et al., 2019) or semantic segmentation (Schmitt et al., 2019), as described in the benchmark datasets provided in TorchGeo (see Stewart et al., 2021 of Table 1). *Sen12MS* is for instance a global dataset including 180,662 patches of Sentinel-1/2 256 x 256 m images and MODIS-derived land cover maps (Schmitt et al., 2019). Another dataset, similar to ours in terms of spatial coverage, is named Seasonal Contrast (*SeCo*) (Mañas et al., 2021) and was released in 2021. It gathers 2.65 km × 2.65 km Sentinel-2 image time-series around about 200 K locations worldwide. Time-series include 5 images separated by approximately 3 months. The objective was to learn an encoder that can be used for a variety of tasks, from land-cover classification to change detection. *SeCo* includes images from all over the world to represent a wide variety of landscapes. Among the currently available and open datasets, our dataset is, to the best of our knowledge, the only one providing monthly image data at so many points worldwide. In order to allow its reuse and the reproducibility of our experiments, the entire dataset is made publicly available with the Zenodo DOI *10.5281/zenodo.4972593*. We also share the scripts that allowed us to create it at https://gitlab.inria.fr/jestopin/sen2patch. In particular, these can be used to collect new image time series at locations other than those covered by our dataset.

### 4.4. Interpretability: In Which Cases Is the Modeling of the Temporal Dynamics the Most Beneficial?

One of the major conclusions of our study is that the regions benefiting the most from a performance gain due to the modeling of the temporal dynamics of satellite images are those with the highest species diversities. This conclusion may seem counterintuitive at first. Indeed, the regions with the highest diversities are often located toward the tropics and are not those with the most pronounced seasonal patterns. Consequently, the image time series in these regions are not expected to be the ones with the strongest temporal signal. However, it is important to understand that the model operates on a global scale with thousands of habitats to discriminate from each other. Whatever the temporal signature of a given habitat, it is a piece of useful information for distinguishing it from other habitats. At the extreme, the temporal signature of a constant habitat throughout the year is a strong marker of that habitat. A study led in Mediterranean natural habitats analyzed habitat discrimination from a variety of multispectral sensors answers simulated from field measurements, including Sentinel-2 (Féret et al., 2015). They showed that multi-temporal acquisitions outperform single data acquisition to discriminate habitats.

The reason for the higher performance gains in high diversity regions is actually more related to the higher model uncertainty in that regions. Species from these regions are indeed those for which there is the least amount of occurrence data available and our study clearly demonstrates that the performance gain is strongly correlated with this variable. In other words, our study shows that the addition of the temporal information allows reducing the model uncertainty related to the lack of occurrence data in high diversity regions. This result appears particularly interesting since habitats with the highest diversity and the rarest species are also the most threatened ones and modeling them is essential to put in place adapted conservation measures.

### 4.5. Key Considerations for Building New Models With Our Method or Using Existing Ones

Our method could be readily applied to other taxonomic groups than the orchids family. The ease and cost of implementation will mainly depend on the geographical distribution of the occurrences of the target taxon. With a family as large and widespread as the orchids, our method requires significant computing resources. Downloading Sentinel-2 tiles to a very large extent demands a lot of storage available (about 100Tb). To keep model training time reasonable, GPUs have to be used too. A computing cluster is more than welcome and the technical requirements can be a limitation for some researchers. However, once the dataset is built and the model is trained, predictions can perfectly be run on standard local machines. To this end, the model built for our study is shared publicly in the same Zenodo repository as the dataset (https://doi.org/10.5281/zenodo.4972593). The new S2 image time-series as input can be used to predict species orchids composition anywhere on earth or to build high-resolution maps of specific orchid species at a global scale. It may also be used for other ecological tasks *via* transfer learning approaches (i.e., keeping unchanged all the weights of the model except those of the last layer dedicated to species classification, Torrey and Shavlik, 2010).

### 4.6. On Temporal and Spatial Biases

In the context of species and habitats distribution modeling in general, a recurrent challenge is a possible mismatch, both in time and space, between the occurrences and the environmental variables (Phillips et al., 2006). As shown in Figure 2B, in particular, a fraction of the occurrences in our dataset date from several decades ago, while the satellite data is from March 2020 to February 2021. If the environment changed since the observation, e.g., because of a housing project or deforestation, the model may learn incorrect relationships. Figure 11A focuses on this particular issue and acknowledges the influence of occurrence observation date on model performances. The top-30 test accuracy is gradually higher on more recent occurrences than older ones. Interestingly, common and rare species predictions seem to respond in the same manner to temporal shifts between predictive habitat data and species observation dates.

Spatial mismatch can also happen because of the occurrences position uncertainty (Shown in Supplementary Figure 1). However, our model being based on convolutional filters, it is highly robust to such spatial shifts until the true occurrence position does not exceed the extent of the input image (here, 640 m × 640 m). Ideally, only occurrences with a position uncertainty of less than 320 m (half of the patch size) should be considered with our method. Figure 11B traduces the impact of test occurrence coordinates uncertainty on model performance. As expected, top-30 accuracy drops when uncertainty is substantial and there is actually very little chance that the predictive data is anywhere near the actual observation place (see performances on *Q*_4_ quartile). Besides, performance on both common and rare species remains almost constant when uncertainty is inferior to the median equal to 707 meters. Thereby, when the maximum uncertainty is of the order of the patch size, the model performs as well as on very precise occurrences. Finally, the *Q*_3_ marked difference of evolution between micro/macro top-30 accuracy could be explained by the following hypothesis: rare species predictions are more affected by a growing coordinates uncertainty than common species because of more locally specific habitat preferences.

In machine learning, such mismatch between labels and predictive data is called *label noise* (Frénay and Verleysen, 2013) and is actively studied (Ghosh et al., 2017; Lee et al., 2018). The strength of our dataset in counteracting this noise is its very large size, as demonstrated by Rolnick et al. (2017). Their work showed that deep learning models can learn correct generalizations even with massively noisy datasets.

At last, the strong spatial bias present in the *DeepOrchidSeries* dataset influences SDMs predictions (Beck et al., 2014). Such bias results from a very uneven sampling effort (Shown in Figure 4C map) and not from orchids distribution. The use of methods to mitigate spatial bias at the cost of occurrence number is a promising direction to exploit *DeepOrchidSeries* (see abovementioned publication). Nonetheless, true understanding of orchids distribution and health will only be reached with significant and uniform observation effort. Having access to constructive and global predictive data is remarkably valuable but not sufficient. Biodiversity hotspots (Myers et al., 2000) urgently need to be sampled with high standards of care to limit human disturbance. Citizen science initiatives are also contributing to enhancing biodiversity monitoring worldwide (Kobori et al., 2016; Affouard et al., 2017).

### 4.7. Perspective 1: Enriching the Input With Other Predictors Informing Orchids Habitats

An exciting future development is to add other relevant predictors to our models. Other image time series like the frequently used bio-climatic variables from WorldClim^6^ or Ecosystem Functional Attributes (EFAs, Arenas-Castro et al., 2018, although not independent since they also are computed from satellite data) would bring Supplementary Materials on species ecological niche. Complementary data like altitude^7^, available global human footprint rasters^8^, soil properties variables^9^ (Batjes et al., 2020), or ecoregions (Olson et al., 2001) would help to crystallize species preferences and vulnerabilities as well.

### 4.8. Perspective 2: Using NN Architectures Designed to Extract Long-Term Temporal Dependencies

An active research avenue concerns adapting neural networks architectures to best analyze satellite image time series with broad temporal and spatial coverages. Recurrent CNNs (RCNNs, Lai et al., 2015) achieve significant performance gain in land-cover classification tasks (Rußwurm and Körner, 2018; Garnot et al., 2019), and we anticipate it should also be relevant for the analysis of species distributions and spatio-temporal dynamics. In our case, we can suggest a hybrid architecture relying on an Inception v3 model to first extract the spatial features at each week or month and then an RNN to encode the temporal dimension over a long period of time. 3D CNNs are another promising candidate architecture but, as pointed out by Garnot et al. (2019), convolutions in the temporal dimension are not well adapted to grasp long-term dependencies and assume a regular sampling of occurrences in time, which we do not have. Lastly, spatio-temporal encoders with temporal attention also merit to be tested when seeing their success on other tasks like satellite time-series segmentation (Garnot and Landrieu, 2021). For now, our CNN architecture is considering the stacked time-series of size twelve as a global temporal context. It was proven suited to grasp the local landscape dynamics yearly and globally improve species relative probability of presence prediction. But with larger time-series, attributing more modeling weight to the temporal dimension will be a must. This seems especially relevant given that predictions of rare species and predictions in very diverse regions benefit the most from the temporal information.

## 5. Conclusion

In this paper, we studied for the first time a worldwide SDM based on high-resolution remote sensing image time series. Therefore, we built and shared a substantial dataset (called *DeepOrchidSeries*) aimed at modeling the distribution of orchids on a global scale from Sentinel-2 data. The spatial structure and phenology of species habitat are captured over a whole year for 999,258 occurrences. We then trained deep-SDMs resting on an Inception v3 architecture whose input was modified to deal with 12 months time-series of RGB+IR images. The analysis of the resulting model reveals that the temporal information contained in the time series enables a strong improvement of the predictive performance compared to a purely spatial model. Thanks to interpretability experiments, we did show that seasonal patterns, in particular, are well captured, resulting in better discrimination of habitats all over the world. We also demonstrated that occurrence-poor species and diversity-rich regions are the ones that benefit the most from this improvement, revealing the importance of habitats, temporal dynamics to characterize biodiversity. We hope that this work will pave the way for even more elaborate spatio-temporal models allowing us to predict future trajectories of ecosystems in the context of rapid changes in habitats.

## Data Availability Statement

- Code is available on the *sen2patch* gitlab: https://gitlab.inria.fr/jestopin/sen2patch.
- Occurrences initial GBIF query is https://doi.org/10.15468/dl.4bijtu (accessed August 2019).
- The dataset and models generated for this study can be found in Zenodo at: https://doi.org/10.5281/zenodo.4972593.

## Author Contributions

JE, MS, PB, FM, and AJ: conceptualization, investigation, methodology, writing–original draft, writing—review, and editing. JE and MS: data curation and software. PB and AJ: funding acquisition. PB, FM, and AJ: project administration. MS, PB, FM, and AJ: supervision. MS, FM, and AJ: validation. JE, FM, and AJ: visualization. All authors contributed to the article and approved the submitted version.

## Funding

The work was supported by the INRIA exploratory action CACTUS Fund.

## Conflict of Interest

The authors declare that the research was conducted in the absence of any commercial or financial relationships that could be construed as a potential conflict of interest.

## Publisher's Note

All claims expressed in this article are solely those of the authors and do not necessarily represent those of their affiliated organizations, or those of the publisher, the editors and the reviewers. Any product that may be evaluated in this article, or claim that may be made by its manufacturer, is not guaranteed or endorsed by the publisher.

## Footnotes

^1^ https://www.copernicus.eu/en

^2^ https://sentinelsat.readthedocs.io/en/stable/

^3^https://scihub.copernicus.eu/, queries and downloads require an activated Scihub Copernicus account.

^4^ https://sentinel.esa.int/web/sentinel/missions/sentinel-2

^5^ http://www.idris.fr/annonces/annonce-jean-zay-eng.html

^6^ http://www.worldclim.org

^7^ https://lpdaac.usgs.gov/products/srtmgl1v003/

^8^https://sedac.ciesin.columbia.edu/data/set/wildareas-v3-2009-human-footprint and 1993 version.

^9^ https://soilgrids.org/
