# Supplementary material for: Deep Species Distribution Modeling From Sentinel-2 Image Time-Series: A Global Scale Analysis on the Orchid Family
Source: Front Plant Sci. 2022 Apr 22;13:839327. doi: 10.3389/fpls.2022.839327 (PMC9072833; doi:10.3389/fpls.2022.839327)
Supplement: Supplementary file 1 [file Data_Sheet_1.pdf]

frontiers

Supplementary Material

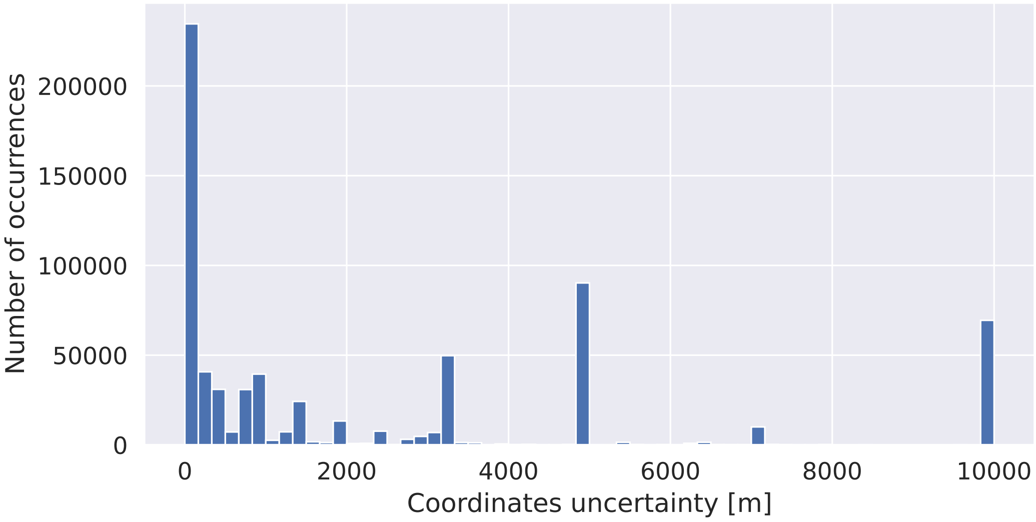

Figure S1. Histogram of occurrences geolocation uncertainty (60 bins). 31% of the 999,248 occurrences associated with satellite data had no uncertainty provided at all and are not represented in this figure. Uncertainty was limited to 10,000 m on the Figure. First quartile is 100 m, median is 850 m and third quartile is 5,000 m. Recent and citizen science occurrences are usually integrating quite precise geolocation (explaining left peaks accumulation) whereas old observations will be less precise. The peak at 5,000 m certainly witnesses an arbitrary uncertainty value attributed to part of the orchids.

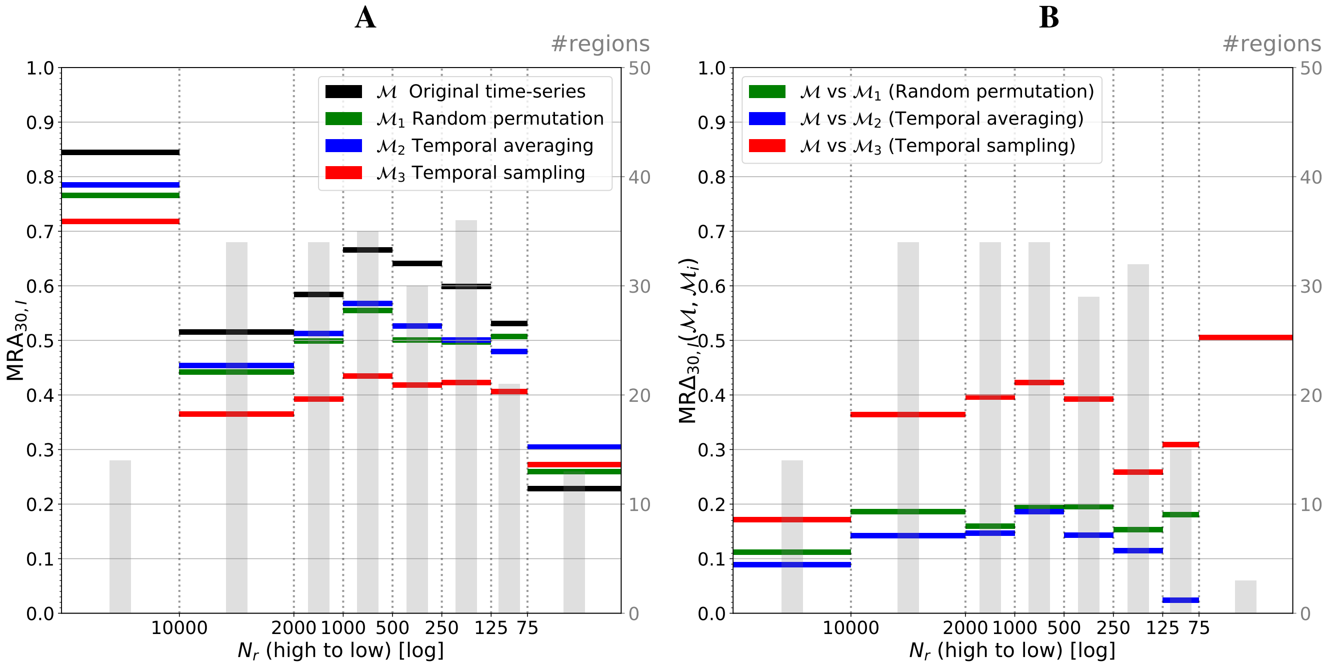

Figure S2. Region top-30 accuracy **(A)** and relative top-30 accuracy change **(B)** averaged per category of number of training occurrences per region N_r. Unlike with categories formed on regions diversity index (Fig. 10A), $\mathrm{MRA}_{30,I}(\mathcal{M})$ does not clearly diminish when regions are including less occurrences (this is not because there is less occurrences that the classification task is harder, the few occurrences can be from very common species). $\mathrm{MR}\Delta_{30,I}(\mathcal{M}, \mathcal{M}_i)$ is not regularly higher in occurrence-poor regions contrary to diversity-rich regions where the predictive data temporal dimension especially help predictions (Fig. 10B).

DATA AVAILABILITY

- Code is available on the *sen2patch* gitlab: *https://gitlab.inria.fr/jestopin/sen2patch*
- Occurrences initial GBIF query is *https://doi.org/10.15468/dl.4bijtu (accessed August 2019)*
- The dataset and models generated for this study can be found in Zenodo at: *https://doi.org/10.5281/zenodo.4972593*.
